# Incorporation of compost and biochar enhances yield and medicinal compounds in seeds of water-stressed *Trigonella foenum-graecum* L. plants cultivated in saline calcareous soils

**DOI:** 10.1186/s12870-024-05182-6

**Published:** 2024-06-12

**Authors:** Ahmed Shaaban, Khaulood A. Hemida, Taia A. Abd El-Mageed, Wael M. Semida, Synan F. AbuQamar, Mohamed T. El-Saadony, Omar A.A.I. Al-Elwany, Khaled A. El-Tarabily

**Affiliations:** 1 Agronomy Department, Faculty of Agriculture, Fayoum University, Fayoum, 63514 Egypt; 2 Botany Department, Faculty of Science, Fayoum University, Fayoum, 63514 Egypt; 3 Soil and Water Department, Faculty of Agriculture, Fayoum University, Fayoum, 63514 Egypt; 4 Horticulture Department, Faculty of Agriculture, Fayoum University, Fayoum, 63514 Egypt; 5 Department of Biology, College of Science, United Arab Emirates University, Al Ain, 15551 United Arab Emirates; 6 Department of Agricultural Microbiology, Faculty of Agriculture, Zagazig University, Zagazig, 44519 Egypt

**Keywords:** Compost-biochar mixtures, Deficit irrigation, Organic soil amendment, Plant-soil-water interaction, Secondary metabolites, Trigonelline

## Abstract

**Background:**

The combination of compost and biochar (CB) plays an important role in soil restoration and mitigation strategies against drought stress in plants. In the current study, the impact of CB was determined on the characteristics of saline calcareous soil and the productivity of fenugreek (*Trigonella foenum-graecum* L.) plants. The field trials examined CB rates (CB_0,_ CB_10_ and CB_20_ corresponding to 0, 10, and 20 t ha^‒1^, respectively) under deficit irrigation [DI_0%_, DI_20%,_ and DI_40%_ receiving 100, 80, and 60% crop evapotranspiration (ETc), respectively] conditions on growth, seed yield (SY), quality, and water productivity (WP) of fenugreek grown in saline calcareous soils.

**Results:**

In general, DI negatively affected the morpho-physio-biochemical responses in plants cultivated in saline calcareous soils. However, amendments of CB_10_ or CB_20_ improved soil structure under DI conditions. This was evidenced by the decreased pH, electrical conductivity of soil extract (ECe), and bulk density but increased organic matter, macronutrient (N, P, and K) availability, water retention, and total porosity; thus, maintaining better water and nutritional status. These soil modifications improved chlorophyll, tissue water contents, cell membrane stability, photosystem II photochemical efficiency, photosynthetic performance, and nutritional homeostasis of drought-stressed plants. This was also supported by increased osmolytes, non-enzymatic, and enzymatic activities under DI conditions. Regardless of DI regimes, SY was significantly (*P* ≤ 0.05) improved by 40.0 and 102.5% when plants were treated with CB_10_ and CB_20_, respectively, as similarly observed for seed alkaloids (87.0, and 39.1%), trigonelline content (43.8, and 16.7%) and WP (40.9, and 104.5%) over unamended control plants.

**Conclusions:**

Overall, the application of organic amendments of CB can be a promising sustainable solution for improving saline calcareous soil properties, mitigating the negative effects of DI stress, and enhancing crop productivity in arid and semi-arid agro-climates.

**Supplementary Information:**

The online version contains supplementary material available at 10.1186/s12870-024-05182-6.

## Background

Fenugreek (*Trigonella foenum-graecum* L.; family *Fabaceae*) is an annual herb indigenous to North Africa and Asia, renowned for its medicinal properties [1]. During the winter season, farmers commonly sow fenugreek seeds alongside fodder crops like clover and barley to enhance the nutritional value of animal feed. Moreover, its tender green leaves and pods are esteemed as edible vegetables for human consumption [2]. Fenugreek seeds contain bioactive compounds such as alkaloids, flavonoids, steroids and saponins along with various secondary metabolites known for their therapeutic benefits, including alkaloidal trigonelline [3–5]. Trigonelline is recognized not only for its potential role as an osmoregulator and osmoprotectant under conditions of salt, drought, and oxidative stresses but also as an inducer of *Nod* genes during the interaction between *Rhizobium* and leguminous plants [6, 7].

Fenugreek encounters significant challenges throughout its life cycle when confronted with environmental stressors. Escalating temperatures and heat stress, attributed to climate change, pose a threat to fenugreek crops, resulting in reduced yield and compromised seed quality [8–10]. As water resources become scarce, fenugreek faces difficulties in sustaining optimal growth and productivity. Although fenugreek prefers slightly acidic to neutral soils, alkaline soils with high carbonate content can impede nutrient availability and hinder plant productivity [11–13]. Moreover, excessive salt levels in soils can restrict water uptake and nutrient transport within fenugreek plants [12].

The anticipated rise in demand for freshwater resources, driven by both shifting global climate patterns and rapid population growth underscores the urgency of addressing significant challenges surrounding freshwater scarcity [14]. Currently, inadequate management of irrigation water exacerbates these shortages, posing substantial obstacles to global food security [15]. To mitigate this issue and ensure food security, the development of water-saving techniques on a global scale is imperative [10, 16]. Deficit irrigation (DI) emerges as a promising strategy to enhance water productivity (WP) without incurring substantial yield loss [17]. As such, DI represents a pivotal cultivation approach for delivering water below full crop-water requirements (evapotranspiration), offering a crucial means of conserving irrigation water, whether applied during specific growth stages or throughout the entire growing season [18, 19].

Studies have consistently demonstrated that any restriction in irrigation water availability is likely to lead to diminished growth and yield of annual crops, including fenugreek [5, 20]. This decline can be attributed to inadequate leaching and high risk of soil salinity, both of which can detrimentally impact crop health and sustainability of irrigation practices [21]. Soils exhibiting an electrical conductivity of soil extract (ECe) exceeding 4 dS m^−1^ are typically indicative of elevated levels of dissolved salts, often termed saline soils [22]. Such soils tend to possess low organic matter (OM) content and elevated pH levels, resulting in compromised nutrient solubility and availability, particularly for phosphorus (P) [23–25]. Generally, soils in arid and semi-arid regions with low OM also exhibit reduced water-holding capacity (WHC) and crop productivity [26]. Saline calcareous soils, prevalent in arid regions, further exacerbate challenges related to soil fertility and nutrient uptake by plants [27].

Among the paramount strategies in conservation agriculture to alleviate the adverse impacts of abiotic stresses, such as heavy metals, drought, and salinity, soil organic amendments stand out [12, 28]. The seasonal incorporation of OM emerges as a prevalent method to mitigate its depletion in arid and semi-arid soils [29], thereby enhancing soil permeability and water retention capacity [28, 30]. Notably, OM amendment not only enhances the physiochemical and biological properties of saline calcareous soils but also furnishes a substantial portion of nutrients essential for improved growth and increased crop yields [10, 31].

Numerous endeavors have been undertaken to explore economical methods for water conservation [32]. For example, various studies have scrutinized the efficacy of OM inputs, such as compost and biochar individually [33–36]. Diacono and Montemurro [31] emphasize that compost represents the final stage of microbial decomposition of organic compounds, characterized by its rich OM and nutritional composition [36].

Compost is recognized for its ability to enrich soil properties, improve crop yields, and enhance WP [37]. Upon incorporation into the soil, microorganisms promptly initiate the decomposition of compost. In contrast, biochar, produced through pyrolysis in oxygen-deprived conditions, exhibits greater durability than compost [38]. The dense carbon (C) structure and aromatic composition of biochar render it more resistant to microbial degradation, thereby augmenting soil OM content [39]. Despite its relatively low nutrient concentration, biochar’s exceptional sorption capacity allows it to retain soil nutrients, mitigating leaching and enhancing water retention [40]. Moreover, the porous nature of biochar not only provides habitats for microorganisms but also fosters microbial activity, thereby bolstering nutrient cycling capabilities [40].

While compost and/or biochar have been investigated independently or in combination for their ability to mitigate various stressors, their combined efficacy as a mixture (CB) in alleviating water stress effects on fenugreek crop productivity in saline calcareous environments remains understudied. In the current study, we proposed that the application of a compost and biochar (CB) mixture would yield synergistic advantages, enhancing soil fertility and improving the growth and yield characteristics of fenugreek plants under DI conditions.

Accordingly, the objectives of this study were: (i) evaluate the impact of using CB as an organic amendment on the physio-chemical properties of soil; (ii) investigate how CB influences morpho-physiological traits, osmoprotectants, photosynthetic efficiency, and enzymatic and non-enzymatic antioxidants in fenugreek plants subjected to drought stress; and (iii) assess seed yield (SY), seed alkaloid and trigonelline content and WP of fenugreek under varying application rates of CB in saline calcareous soil, both with full and deficit irrigation. Overall, the findings of this study demonstrate that incorporating CB as a soil amendment can enhance soil quality and improve the yield of fenugreek plants, particularly under conditions of drought stress in arid and semi-arid regions.

## Methods

### Site description

Two trials were performed in the open field during the growing seasons of 2021–2022 and 2022–2023 in the experimental farm of the Faculty of Agriculture, Fayoum, Egypt (29°17’38” N 30°54’55” E). The climate in the local area is considered arid [41], and the soil is a sandy loam that is saline, calcareous, siliceous, and hyperthermic. It lies between (0.5 and 0.8 m deep) [42].

### Basic soil characteristics

Soil pH was assessed in saturated soil-water paste using LI-120, Digital PH Meter (Elico, Sanathnagar, Hyderabad, Telangana, India), and ECe (dS m^−1^) was measured in saturated soil-water paste extract using CM25 conductivity meter (model 3200, YSI, Inc., Yellow Springs, Ohio, USA) according to Page et al. [43]. Total CaCO_3_ content was determined volumetrically using the Collin’s Calcimeter method, whereas OM content was measured with the wet combustion method [43].

Available N in the soil was measured using the technique of Stanford et al. [44]. Available P was extracted by 0.5 N of NaHCO_3_ solution at pH 8.5 as shown by Olsen et al. [45]. The ratio of soil: extract was 1:20, and the extraction time was 30 min of continuous shaking. After the extract had been filtered, an atomic absorption spectrophotometer was used to calculate the extracted P (Perkin-Elmer Model 3300, Glenbrook, Stamford, CT, USA) [46]. After shaking the soil sample with a 1 N C_2_H_7_NO_2_ solution for 30 min, the amount of available K was calculated using flame photometry Model 52-A (Perkin-Elmer) [43]. Bulk density (BD) was determined using the cylinder method [47].

Total porosity (TP) was calculated using particle density (γ_S_) and the dry BD (γ_d_) values by the following equation:

$$\mathrm{TP}=\left(\gamma S\;-\;\gamma d\;/\;\gamma S\right)\times100$$

Water holding pores (WHP; 8.62 μm–0.19 μm) and useful pores (UP; < 0.19 μm) were determined by measuring both volumetric water content (θ) and matric potential or suction (ψ_m_).

They were determined in the laboratory using a tension table and pressure plate. A flat porous surface was prepared at one end of each core sample to ensure hydraulic contact with the tension table. The samples were then placed on the saturated surface of the tension table, after which they were subjected to different suctions. The samples were weighed after the equilibrium at each successive suction [47]. Field capacity (θ_Fc_) was calculated using the tension table at a tension of 0.33 bar. Available water (AW) was estimated by the difference in water content between θ_Fc_ and permanent wilting point (PWP) as follows:

$$\mathrm{AW}={\mathrm\theta}_{\mathrm{Fc}}-\mathrm{PWP}$$

The physiochemical characteristics of the tested soil were: ECe = 8.51 dS m^−1^, pH = 7.96, total N = 1.3 g kg^−1^, extractable P = 3.37 mg kg^−1^, extractable K = 39.52 mg kg^−1^, OM = 0.92%, CaCO_3_ = 16.52%, BD = 1.56 kg m^−3^, and soil moisture content at θ_Fc_ and wilting point = 18.49% and 8.11%, respectively [48]. The meteorological data of the experimental site are shown in Table (S1).

### Treatments and agronomic management

Randomized complete block design (RCBD) with the split plot arrangement was used in this experiment. Treatments were divided into three water applications and three compost-biochar mixture (CBM) rates. Irrigated water was applied as a percentage of crop evapotranspiration (ETc), representing three treatments: full irrigated FI (DI_0%_) = 100%, DI_20%_ = 80% and DI_40%_ = 60% of ETc, while CB mixtures were CB_0_ = 0 t ha^−1^ (control), CB_10_ = 10 t ha^−1^ (5 t ha^−1^ compost + 5 t ha^−1^ biochar) and CB_20_ = 20 t ha^−1^ (10 t ha^−1^ compost + 10 t ha^−1^ biochar).

Biochar was obtained by slow pyrolysis of wood of *Mangifera indica* in a biochar kiln at a temperature range of 350–450 °C. The used compost was prepared from 25 kg of *Pelargonium graveolens* waste material (25%), 0.5 kg of rice straw (0.5%) to provide some free air pores and maintain the aerobic conditions, 0.5 kg of K-humate (0.5%), and 12 kg of each cattle manure (24%) and green Egyptian clover plants (24%) as a N element source [49]. All ingredients were well blended and then composted in a pile measuring 25 × 2 × 1.6 m (length x width x height). The pile was turned over four times a month during the bio-oxidation stage and regularly sprinkled with water to maintain a 60% (*v/w*) wet level. The composting process continued from April 20 to July 20, up to the intermix maturation of all composted materials.

Irrigation treatments were set as the main plots, while CB treatments were randomly distributed in the sub-plots. CB was applied to the soil three weeks before planting fenugreek seeds. Table (S2) lists the characteristics of CB employed in the current experiments. Nine treatments were replicated three times, and the entire experimental plots were 27. The fenugreek seeds were acquired from the Department of Medicinal and Aromatic Plants of the Egyptian Ministry of Agriculture in Giza, Egypt. Seeds (120 kg ha^−1^) of fenugreek (cv. Giza 2) were manually planted on October 14, 2021 (Growing season 2021–2022) and October 17, 2022 (Growing season 2022–2023) in beds (15 m in length × one-meter width). Each of the 15 m^2^ bed areas contained four planting rows (20 cm apart) and 5–7 cm spacing between plants within rows.

In this experiment, irrigation with two dripper lines per row, one on each side, were placed about 0.5 m apart. The drippers along the lines were spaced at 1.7 m accordingly. During soil preparation, the recommended rate of nitrogen (N), P, and K was 50, 75, and 120 kg ha^−1^, respectively. N was added in the form of ammonium nitrate (33.5% N), P in the form of calcium super phosphate (15.5% P_2_O_5_), and K in the form of potassium sulfate (48% K_2_O). Appropriate agronomic management and pest control for the fenugreek crop was carried out following the recommendation of the Egyptian Agricultural Research Center, Giza, Egypt.

### Irrigation water applied (IWA) and WP

The daily reference evapotranspiration (ETo) was calculated according to the technique of FAO Penman-Monteith equation [48]. As shown in the following equation, ETc was calculated using the ETo and the crop coefficient (Kc). The flow rate of the drip irrigation system was 3 L ha^−1^. The ETc (mm d^−1^) was estimated [48], as the following:

$$\mathrm{ETc}=\mathrm{Kc}\times{\mathrm E}_{\mathrm{pan}}\times{\mathrm K}_{\mathrm{pan}}$$

Where, Kc = crop coefficient. According to [50, 51], the stage-specific Kc values of fenugreek crop at the initial stage, mid-stage, and late-season stage were 0.69, 1.02, and 0.87, respectively. Epan = evaporation from the Class A pan (mm d^−1^) and Kpan = the pan evaporation coefficient.

The WP was computed using the formula [19] given below:

$$\mathrm{WP}=\frac{\mathrm{Seed}\;\mathrm{yield}\;\left(\mathrm{kg}\;\mathrm{ha}^{-1}\right)}{\mathrm{Water}\;\mathrm{applied}\;\left(\mathrm m^3\;\mathrm{ha}^{-1}\right)}$$

### Measurements of plant growth and key physiological indices

The measured traits of the fenugreek plants were taken at the full blooming stage (90 days after sowing). These traits were plant height (PH; cm), root length (RL; cm), number of branches and leaves plant^−1^, and dry weight (DW; g plant^−1^).

The chlorophyll *a* fluorescence parameters of fenugreek plants were measured with a portable chlorophyll fluorometer (Handy-PEA, Hansatech, UK). For each treatment, the measurements were performed on fully expanded leaves of five plants in the morning (10:00-1100 AM) after dark adaptation for 20 min. Chlorophyll fluorescence was induced by applying a pulse of saturating red light (650 nm). This measurement yielded the values of the minimum fluorescence (F_0_), maximum fluorescence (F_m_), while the maximal efficiency of PSIΙ photochemistry (*F*_*v*_*/F*_*m*_) and the potential activity of PSII (*F*_*v*_*/F*_*0*_) were calculated according to Maxwell and Johnson [52]. The photosynthetic performance index (PI) was also determined as reported previously [53].

The relative water content (RWC%) and membrane stability index (MSI%) values were calculated following the methods given by [54, 55], respectively. A chlorophyll meter (SPAD-502, Minolta, Osaka, Japan) was used to determine the relative chlorophyll content (SPAD_chlorophyll_).

### Measurements of SY and yield-related attributes

Five plants were randomly harvested from each experimental plot on April 17 and 19 of the 2022 and 2023 seasons to determine the number of pods plant^−1^, SY plant^−1^ (g), and seed index (1000-seed weight; SI; g). All fenugreek plants in each sub-plot were manually harvested, sun-dried for two days, and then weighed along with the five fenugreek plants sampled before to estimate biological yield (BY; t ha^−1^), SY (t ha^−1^) based on 12% moisture, as well as seed harvest index (SHI) by dividing SY by BY in t ha^−1^.

### Assays of oxidative stress indicators, osmoprotectants, and non-enzymatic antioxidants

To assay oxidative stress, hydrogen peroxide (H_2_O_2_; µmol mg^−1^ FW) was determined as previously described [56]. Malondialdehyde (MDA) was also tested in plant tissues [57] to determine the extent of lipid peroxidation. An attenuation coefficient of 155 mM^−1^ cm^−1^ was used to compute MDA concentration in µmol mg^−1^ FW. Total soluble sugars (TSS; mg g^−1^ DW) were extracted [58] and measured using a UV-160 A UV Visible Recording Spectrometer (Bausch and Lomb analytical systems divisions, Rochester, USA) at 625 nm. Free proline concentration (FProC; mg g^−1^ DW) was rapidly estimated at 520 nm using the colorimetric approach [59].

By using the methanolic solvent [60], total phenolics (TPhs; mg g^−1^ DW) were extracted from dried tissues, and the Folin–Ciocalteau phenol method [61] was used for phenolic determination. Soluble proteins were extracted using Moore’s method [62] and extraction yield (%) was determined [63]. The reduced glutathione (GSH) and ascorbic acid (AsA) contents in fresh leaf tissues of fenugreek were determined using the techniques previously outlined [64, 65].

### Enzymatic antioxidants and 2,2-diphenyl-1- picrylhydrazyl (DPPH)-scavenging activity

Fresh leaf tissue (0.5 g) was used for superoxide dismutase (SOD), catalase (CAT), glutathione reductase (GR), and ascorbate peroxidase (APX) extraction. Samples were homogenized in 0.1 M ice-cold phosphate buffer (pH = 7.5) containing 0.5 mM EDTA with pre-chilled pestle and mortar. Each homogenate was transferred to centrifuge tubes and centrifuged at 4ºC in a Beckman refrigerated centrifuge for 15 min at 15,000 × *g* and the supernatant was used for the enzyme activity assay. The concentration of the extracted protein was determined [63]. The activity of SOD (EC 1.15.1.1) was assessed by recording the inhibition of cytochrome reduction in nitroblue tetrazolium at 540 nm [66]. CAT (EC 1.11.1.6) was determined by measuring the decomposition rate of H_2_O_2_ at 240 nm [67]. GR (EC 1.6.4.1) was determined by measuring the oxidation of NADPH at 340 nm; whereas ascorbate peroxidase (APX; EC 1.11.1.11) was assessed by monitoring the rate of ascorbate oxidation at 290 nm (E = 2.8 mM^−1^ cm^−1^) [68]. DPPH radical-scavenging activity (DPPH RSA) of all the extracts was investigated using DPPH free radical method [69].

### Measurements of total alkaloid and trigonelline content

For trigonelline determination, one gram of powdered dried seeds of fenugreek was weighed and mixed with one gram of magnesium oxide (MgO) and 20 ml of distilled water. The mixture was incubated in a water bath at 100 °C for 20 min. After cooling, the mixture was filtered through Whatman paper number 1 (Cytiva, Buckinghamshire, United Kingdom), and its volume was brought to 25 ml with distilled water. The absorbance of the solutions was measured in UV-vis spectrophotometer apparatus at 268 nm. A standard curve was used to calculate the sample’s trigonelline content, which was represented as mg g^−1^ DW [70, 71].

The preparation of solution and extraction procedures were as recommended by [72]. Extracts were collected in a 10-ml volumetric flask and diluted with chloroform. The absorbance of the complex in chloroform was measured at 470 nm.

### Determination of leaf mineral contents

The macro-elements (N, P, K^+^, Ca^2+^, and Na^+^) content of fenugreek leaves was determined by drying and grinding the leaves into a powder. The dried samples were subjected to a digestion process using a solution of HClO_4_ and H_2_SO_4_ (at 1:3 v/v, respectively). N content was assessed using micro-Kjeldahl equipment (Ningbo Medical Instruments Co., Ningbo, China) [73]. Molybdenum blue, diluted H_2_MoO_7_S, and 8% (w/v) NaHSO_3_-H_2_SO_4_ were used as standard reagents for quantifying P [74]. K^+^, Ca^2+^, and Na^+^ contents were measured using a Perkin-Elmer Model 52-A Flame Photometer [75].

### 2.11 Statistical analysis

Before the analysis of variance (ANOVA), Shapiro-Wilk normality and Bartlett homogeneity tests were used to explore if the dataset of each variable was normal and whether the error variances of both seasons were homogeneous. The outputs of these two tests, as pre-ANOVA assumptions, showed that all variables are statistically acceptable to perform ANOVA and Duncan multiple comparison tests (with a 5% confidence interval). A split-plot RCBD was used to base the combined analysis for the two experimental seasons ANOVA [76] and with three replicates using INFOSTAT computer software (v.2019 statistical package, Córdoba University, Córdoba, Argentina) [77].

## Results

### Soil hydro-physico-chemical properties in response to CB mixture

The main hydro-physico-chemical characteristics of soil were markedly (*P* ≤ 0.05) affected by the amendments of CB_10_ or CB_20_ (Table 1). Soil pH, ECe, and BD for each of the CB_10_- and CB_20_-amended soils were 3.0 and 4.8%, 20.4 and 28.2%, and 1.9 and 5.1%, respectively, significantly (*P* ≤ 0.05) lower than without CB amendment. However, CB_10_- and CB_20_-amended soils exhibited a progressive improvement in OM by 26.1 and 53.3%, soil N by 100.0 and 450.0%, P by 62.6 and 92.0%, and K by 12.7 and 44.7%, WHP by 35.8 and 59.3%, UP by 53.7 and 85.9%, TP by 15.0 and 22.1%, and soil water content at FC by 30.3 and 31.4% and available water (AW) by 43.3 and 48.8%, respectively, as compared to an unamended saline calcareous soil (Table 1).

**Table 1:** Effect of the application of CB mixture on soil hydro-physico-chemical properties across the 2021–2022 and 2022–2023 cropping seasons

CB (t ha^−1^)	Soil hydro-physical properties					
	BD	WHP	UP	TP	θ_Fc_	AW
	(g cm^−3^)	(%)				
0 (CB_0_)	1.56 ± 0.12a	10.74 ± 0.12c	11.41 ± 0.12c	32.1 ± 1.2c	18.49 ± 1.1b	9.71 ± 0.87b
10 (CB_10_)	1.53 ± 0.11b	14.58 ± 0.75b	17.54 ± 0.75b	36.9 ± 1.3b	24.1 ± 1.3a	13.91 ± 0.99a
20 (CB_20_)	1.48 ± 0.11c	17.11 ± 1.2a	21.21 ± 1.2a	39.2 ± 1.7a	24.3 ± 1.4a	14.45 ± 1.21a
CB (t ha^−1^)	Soil chemical properties					
	EC_e_ (dS m^−1^)	Soil pH	OM	N	P	K
			(%)		(mg kg^−1^ soil)	
0 (CB_0_)	8.51 ± 0.88a	7.96 ± 1.12a	0.92 ± 0.11c	0.004 ± 0.00c	3.37 ± 0.33c	39.52 ± 3.51c
10 (CB_10_)	6.77 ± 1.13b	7.72 ± 0.98b	1.16 ± 0.15b	0.008 ± 0.00b	5.48 ± 0.25b	44.52 ± 3.62b
20 (CB_20_)	6.11 ± 0.97c	7.58 ± 1.21c	1.41 ± 0.12a	0.022 ± 0.00a	6.47 ± 0.32a	57.18 ± 4.2a

Each value indicates mean ± standard error (*n* = 3). Means values in each column for DI, CB, or DI × CB levels followed by the same lower-case letter in each column are not significantly different according to the Duncan test (*P* ≤ 0.05). *CB* compost and biochar, *BD* bulk density, *WHP* water holding pores, *UP* useful pores, *TP* total porosity, *θ*_*Fc*_ field capacity, *AW* available water, *EC*_*e*_electrical conductivity of soil extract, *OM* organic matter, *N* nitrogen, *P* phosphorus, *K* potassium

### Growth attributes and dry matter of fenugreek plants

Characteristics of shoot-root formation in fenugreek plants cultivated in salty calcareous soil were negatively affected by the reduction of soil moisture conditions. Drought stress at DI_20%_ and DI_40%_ levels significantly (*P* ≤ 0.05) decreased PH by 15.8 and 27.4%, number of branches plant^−1^ by 24.0 and 45.2% and number of leaves plant^−1^ by 37.2 and 56.0%, RL by 15.5 and 27.1%, and dry matter plant^−1^ by 32.5 and 54.5%, respectively, when compared to FI level (Table 2). Adding CB to saline calcareous soil at a rate of 10 or 20 t ha^−1^ pronouncedly (*P* ≤ 0.05) improved the PH by 34.9 or 77.8%, number of branches plant^−1^ by 46.2 or 88.0%, number of leaves plant^−1^ by 90.2 or 154.3%, RL by 11.9 or 23.3%, and dry matter plant^−1^ by 97.8 or 237.8%, respectively, compared to unamended (CB_0_) control fenugreek plants (Table 2).

**Table 2:** Effect of the application of CB mixture along with different DI levels on shoot-root growth attributes of fenugreek (*Trigonella foenum-graecum* L.) plants grown under saline calcareous soil conditions in S_I_ and S_II_ growing seasons

Treatment		PH (cm)	Number of branches plant^−1^	Number of leaves plant^−1^	RL (cm)	Dry matter (g plant^−1^)
Season		*	NS	**	**	*
S_I_		29.7 ± 1.6b	7.26 ± 0.5a	77.0 ± 8.1b	15.2 ± 0.6b	15.9 ± 1.8b
S_II_		39.7 ± 2.3a	7.70 ± 0.5a	96.9 ± 9.2a	20.3 ± 0.7a	22.1 ± 2.7a
DI		**	**	**	**	**
FI (DI_0%_)		40.5 ± 2.8a	9.72 ± 0.6a	126.2 ± 11.7a	20.7 ± 1.0a	26.8 ± 3.4a
DI_20%_		34.1 ± 2.3b	7.39 ± 0.5b	79.2 ± 7.9b	17.5 ± 0.8b	18.1 ± 2.1b
DI_40%_		29.4 ± 2.3c	5.33 ± 0.4c	55.5 ± 4.4c	15.1 ± 0.8c	12.2 ± 1.7c
CB (t ha^−1^)		**	**	**	**	**
0 (CB_0_)		25.2 ± 1.4c	5.17 ± 0.4c	47.9 ± 3.2c	15.9 ± 1.0c	9.0 ± 1.0c
10 (CB_10_)		34.0 ± 1.7b	7.56 ± 0.5b	91.1 ± 9.2b	17.8 ± 1.0b	17.8 ± 1.4b
20 (CB_20_)		44.8 ± 2.4a	9.72 ± 0.6a	121.8 ± 10.7a	19.6 ± 0.9a	30.4 ± 2.9a
DI × CB		NS	**	**	NS	**
FI (DI_0%_)	CB_0_	30.0 ± 1.9a	6.67 ± 0.3c	62.8 ± 3.4e	18.7 ± 2.0a	13.1 ± 1.2e
	CB_10_	39.0 ± 2.6a	9.83 ± 0.5b	141.7 ± 5.6b	21.0 ± 1.5a	23.3 ± 1.7c
	CB_20_	52.5 ± 4.2a	12.67 ± 0.3a	174.2 ± 6.7a	22.5 ± 1.5a	44.0 ± 4.1a
DI_20%_	CB_0_	25.5 ± 2.0a	5.14 ± 0.3d	45.7 ± 2.8f	15.7 ± 1.3a	8.9 ± 1.0f
	CB_10_	32.8 ± 2.5a	7.33 ± 0.2c	73.2 ± 3.9d	17.3 ± 1.3a	18.0 ± 1.6d
	CB_20_	44.0 ± 3.2a	9.67 ± 0.4b	118.7 ± 8.5c	19.5 ± 1.2a	27.5 ± 2.3b
DI_40%_	CB_0_	20.0 ± 1.5a	3.67 ± 0.5e	35.3 ± 3.1 g	13.3 ± 1.3a	4.9 ± 0.8 g
	CB_10_	30.2 ± 2.9a	5.50 ± 0.2d	58.5 ± 4.9e	15.0 ± 1.3a	12.0 ± 1.7ef
	CB_20_	38.0 ± 3.2a	6.83 ± 0.5c	72.7 ± 4.5d	16.8 ± 1.4a	19.7 ± 1.5d

Each value indicates mean ± standard error (*n* = 3). Means values in each column for DI, CB, or DI × CB levels followed by the same lower-case letter in each column are not significantly different according to the Duncan test (*P ≤* 0.05). *CB* compost and biochar, *DI* deficit irrigation, *PH* plant height, *RL *root length, S_I_ and S_II_, 2021–2022 and 2022–2023 growing seasons, respectively. * and **, differences at *p* ≤ 0.05 and 0.01 probability level; *NS* no significant difference *P* ≤ 0.05. *FI* full irrigation (DI_0%_) control received 100% of crop evapotranspiration (ETc), and DI_20%_ and DI_40%_, received 80% and 60% ETc, respectively; CB_0_, CB_10_ and CB_20_, CB mixture (1:1; w/w) at 0, 10, and 20 t ha^−1^, respectively

The interactive effect of DI levels and CB rates showed considerable improvements in the number of branches plant^−1^, number of leaves plant^−1^, and dry matter plant^−1^ of fenugreek plants under saline calcareous soil conditions. The FI × CB_20_-treated fenugreek plants showed the maximum increases in number of branches plant^−1^ by 245.2%, number of leaves plant^−1^ by 393.5%, and dry matter plant^−1^ by 798.0%, compared to DI_40%_ × CB_0_-treated plants displaying the minimum mean values of these characteristics (Table 2).

### Cell integrity and leaf photosynthetic efficiency

DI strategies and CB application rates individually or in combinations (DI × CB) significantly (*P* ≤ 0.05) affected cell integrity and leaf photosynthetic efficiency of fenugreek plants in terms of RWC, MSI, SPAD_chlorophyll_, PSIΙ photochemical efficiency (*F*_*v*_*/F*_*m*_) and PSII potential photochemical activity (*F*_*v*_*/F*_*0*_) and PI (Table 3). Compared to FI fenugreek plants, drought stress at DI_20%_ or DI_40%_ markedly (*P* ≤ 0.05) decreased RWC by 16.8 or 31.2%, MSI by 6.5 or 20.4%, SPAD by 9.5 or 41.9%, *F*_*v*_*/F*_*m*_ by 3.6 or 14.3%, *F*_*v*_*/F*_*0*_ by 11.2 or 30.4%, and PI by 28.2 or 60.0%, respectively (Table 3).

**Table 3:** Effect of the application of CB mixture along with different DI levels on RWC, MSI, SPAD, *F*_*v*_*/F*_*m*_, *F*_*v*_*/F*_*0*_, and PI of fenugreek (*Trigonella foenum-graecum* L.) plants grown under saline calcareous soil conditions in S_I_ and S_II_ growing seasons

Treatment		RWC (%)	MSI	SPAD	F_v_ /F_m_	F_v_ /F_0_	PI
Season		NS	NS	*	NS	NS	NS
S_I_		66.3 ± 2.7a	62.8 ± 2.2a	47.3 ± 3.4b	0.79 ± 0.01a	4.75 ± 0.2a	12.86 ± 1.3a
S_II_		71.7 ± 2.6a	63.2 ± 2.4a	52.7 ± 3.4a	0.78 ± 0.01a	4.82 ± 0.2a	12.92 ± 1.3a
DI		**	**	**	**	**	**
FI (DI_0%_)		82.1 ± 2.1a	69.2 ± 2.7a	60.3 ± 3.2a	0.84 ± 0.01a	5.56 ± 0.1a	18.26 ± 1.3a
DI_20%_		68.3 ± 2.4b	64.7 ± 2.5b	54.6 ± 3.3b	0.81 ± 0.01b	4.94 ± 0.2b	13.11 ± 1.1b
DI_40%_		56.5 ± 2.2c	55.1 ± 2.2c	35.0 ± 3.4c	0.72 ± 0.02c	3.87 ± 0.3c	7.30 ± 1.0c
CB (t ha^−1^)		**	**	**	**	**	**
0 (CB_0_)		61.1 ± 3.0c	51.7 ± 1.8c	33.1 ± 2.4c	0.74 ± 0.02c	3.84 ± 0.3c	8.03 ± 1.0c
10 (CB_10_)		67.9 ± 2.9b	63.1 ± 1.5b	53.5 ± 3.9b	0.79 ± 0.01b	4.98 ± 0.1b	12.71 ± 1.3b
20 (CB_20_)		78.0 ± 2.8a	74.2 ± 2.1a	63.3 ± 2.1a	0.83 ± 0.01a	5.53 ± 0.1a	17.93 ± 1.3a
DI × CB		NS	*	**	NS	**	NS
FI (DI_0%_)	CB_0_	75.2 ± 2.4a	58.9 ± 1.8d	43.1 ± 1.4e	0.80 ± 0.01a	5.14 ± 0.2bc	12.19 ± 0.8a
	CB_10_	81.2 ± 2.0a	65.6 ± 2.0c	66.3 ± 1.1b	0.84 ± 0.01a	5.46 ± 0.1b	18.79 ± 0.9a
	CB_20_	89.9 ± 3.6a	83.0 ± 1.9a	71.7 ± 2.5a	0.87 ± 0.01a	6.07 ± 0.1a	23.96 ± 0.7a
DI_20%_	CB_0_	59.9 ± 2.9a	51.9 ± 1.8e	35.8 ± 0.9f	0.76 ± 0.01a	4.00 ± 0.1d	8.15 ± 1.0a
	CB_10_	67.3 ± 3.5a	66.9 ± 1.8c	62.6 ± 1.6c	0.81 ± 0.02a	5.16 ± 0.1bc	13.27 ± 1.0a
	CB_20_	77.9 ± 1.9a	75.3 ± 1.9b	65.5 ± 1.4b	0.84 ± 0.01a	5.65 ± 0.1ab	17.86 ± 1.1a
DI_40%_	CB_0_	48.2 ± 2.4a	44.2 ± 1.8f	20.6 ± 2.2 h	0.65 ± 0.02a	2.39 ± 0.2e	3.60 ± 0.8a
	CB_10_	55.2 ± 1.9a	57.0 ± 1.8de	31.6 ± 1.6 g	0.72 ± 0.01a	4.32 ± 0.2d	6.34 ± 0.9a
	CB_20_	66.3 ± 2.6a	64.2 ± 2.0c	52.9 ± 0.9d	0.79 ± 0.02a	4.89 ± 0.1c	11.56 ± 0.9a

Each value indicates mean±standard error (*n*=3). Means values in each column for DI, CB, or DI × CB levels followed by the same lower-case letter in each column are not significantly different according to the Duncan test (*P≤*0.05). *CB*, compost, and biochar, *DI* deficit irrigation, *RWC* relative water content, *MSI* membrane stability index, *SPAD* soil–plant-analysis development chlorophyll; *F*_v_ */F*_m_ and *F*_v_ */F*_0_, chlorophyll fluorescence, *PI* performance index; S_I_ and S_II_, 2021-2022 and 2022-2023 growing seasons, respectively. * and **, differences at *p* ≤ 0.05 and 0.01 probability level; *NS* no significant difference *P*≤0.05. *FI* full irrigation (DI_0%_) control received 100% of crop evapotranspiration (ETc), and DI_20%_and DI_40%_, received 80% and 60% ETc, respectively; CB_0_, CB_10_ and CB_20_, CB mixture (1:1; w/w) at 0, 10, and 20 t ha^-1^, respectively

Under saline calcareous soil conditions, the application rate of 10 or 20 t CB ha^−1^ significantly (*P* ≤ 0.05) improved all the traits mentioned above by 11.1 or 27.7%, 22.1 or 43.5%, 61.6 or 91.2%, 6.8 or 12.2%, 29.7 or 44.0%, and 58.3 or 123.3%, respectively, compared to CB_0_-treated plants (control) (Table 3). When the interaction of DI × CB was applied, the best results for cell integrity and leaf photosynthetic efficiency were obtained at FI × CB_20_ and DI_20%_ × CB_20_ interactions, which significantly (*P* ≤ 0.05) improved MSI by 87.8 and 70.4%, SPAD by 248.1 and 218.0%, and *F*_*v*_*/F*_*0*_ by 154.0 and 136.4%, respectively, compared to DI_40%_ × CB_0_ interaction over the two growing seasons (Table 3).

### Yield and yield-related attributes and WP

DI stress induced by DI_20%_ and DI_40%_ levels also negatively affected fenugreek yield and yield-related attributes but positively affected WP (Table 4). There were significant (*P* ≤ 0.05) decreases in the number of pods plant^−1^, SY plant^−1^, SI, SHI, BY, and SY by 26.4 or 50.5%, 36.8 or 58.8%, 20.7 or 33.0%, 10.3 or 18.7%, 1.9 or 13.7%, and 12.6 or 30.1%, respectively, in plants supplied with DI_20%_ or DI_40%_ compared to FI plants (Table 4). The WP; however, significantly (*P* ≤ 0.05) increased by 10.0 or 16.7%, respectively by the same DI treatments (Table 4).

**Table 4:** Effect of the application of CB mixture along with different DI levels on SY and yield-related attributes and water productivity (WP) of fenugreek (*Trigonella foenum-graecum* L.) plants grown under saline calcareous soil conditions across (S_I_) 2021–2022 and (S_II_) 2022–2023 seasons

Treatment		Number of pods plant^−1^	SY plant^−1^	SI	SHI (%)	BY	SY	WP (kg m^−3^)
			(g)			(t ha^−1^)		
Season		*	NS	NS	*	NS	*	**
S_I_		26.6 ± 2.6b	5.61 ± 0.6a	14.8 ± 0.8a	38.2 ± 1.2b	4.39 ± 0.19a	1.71 ± 0.11b	0.62 ± 0.04b
S_II_		30.8 ± 2.9a	6.07 ± 0.6a	14.5 ± 0.8a	39.0 ± 1.1a	4.62 ± 0.19a	1.83 ± 0.11a	0.69 ± 0.04a
DI		**	**	**	**	**	**	**
FI (DI_0%_)		38.6 ± 3.7a	8.57 ± 0.6a	17.9 ± 1.1a	42.7 ± 1.1a	4.75 ± 026a	2.06 ± 0.15a	0.60 ± 0.05c
DI_20%_		28.4 ± 2.7b	5.42 ± 0.5b	14.2 ± 0.5b	38.3 ± 1.5b	4.66 ± 0.16a	1.80 ± 0.12b	0.66 ± 0.04b
DI_40%_		19.1 ± 1.9c	3.53 ± 0.4c	12.0 ± 0.4c	34.7 ± 0.9c	4.10 ± 0.24b	1.44 ± 0.10c	0.70 ± 0.05a
CB (t ha^−1^)		**	**	**	**	**	**	**
0 (CB_0_)		14.3 ± 1.0c	3.70 ± 0.4c	11.8 ± 0.4c	33.6 ± 1.0c	3.58 ± 0.12c	1.20 ± 0.05c	0.44 ± 0.01c
10 (CB_10_)		31.1 ± 2.5b	5.44 ± 0.5b	14.1 ± 0.5b	38.6 ± 0.9b	4.33 ± 0.11b	1.68 ± 0.06b	0.62 ± 0.02b
20 (CB_20_)		40.8 ± 2.8a	8.38 ± 0.7a	18.1 ± 1.1a	43.4 ± 1.3a	5.60 ± 0.13a	2.43 ± 0.09a	0.90 ± 0.02a
DI × CB		**	**	**	*	**	**	**
FI (DI_0%_)	CB_0_	18.2 ± 1.2e	5.71 ± 0.2c	13.5 ± 0.4d	38.0 ± 1.0c	3.64 ± 0.11d	1.38 ± 0.05e	0.40 ± 0.02 h
	CB_10_	44.0 ± 1.1b	8.01 ± 0.2b	16.1 ± 0.4b	42.7 ± 1.0b	4.51 ± 0.14c	1.92 ± 0.06c	0.56 ± 0.02f
	CB_20_	53.7 ± 1.8a	12.01 ± 0.3a	24.0 ± 0.4a	47.4 ± 1.5a	6.11 ± 0.19a	2.88 ± 0.04a	0.84 ± 0.02c
D_20%_	CB_0_	15.2 ± 0.9f	3.50 ± 0.2d	11.6 ± 0.2e	32.0 ± 1.3d	3.96 ± 0.16d	1.26 ± 0.02f	0.46 ± 0.01 g
	CB_10_	29.0 ± 1.8d	4.97 ± 0.2c	14.5 ± 0.2c	37.1 ± 0.8c	4.68 ± 0.11c	1.74 ± 0.04d	0.64 ± 0.02e
	CB_20_	41.2 ± 2.1c	7.79 ± 0.4b	16.5 ± 0.5b	45.6 ± 1.4ab	5.34 ± 0.22b	2.42 ± 0.04b	0.89 ± 0.02b
DI_40%_	CB_0_	9.5 ± 0.9 g	1.90 ± 0.1e	10.3 ± 0.2f	30.9 ± 1.4d	3.13 ± 0.20e	0.96 ± 0.03 g	0.47 ± 0.02 g
	CB_10_	20.2 ± 1.2e	3.36 ± 0.2d	11.8 ± 0.3e	36.1 ± 1.5c	3.81 ± 0.12d	1.37 ± 0.04e	0.67 ± 0.02d
	CB_20_	27.5 ± 1.4d	5.33 ± 0.2c	13.8 ± 0.5 cd	37.3 ± 0.6c	5.36 ± 0.06b	2.00 ± 0.02c	0.97 ± 0.02a

Each value indicates mean ± standard error (*n* = 3). Means values in each column for DI, CB, or DI × CB levels followed by the same lower-case letter in each column are not significantly different according to the Duncan test (*P ≤* 0.05). *CB* compost and biochar, *DI* deficit irrigation, *BY* biological yield, *SY* seed yield, *WP* water productivity, *SI* seed index, *SHI* seed harvest index; S_I_ and S_II_, 2021–2022 and 2022–2023 growing seasons, respectively. * and **, differences at *P* ≤ 0.05 and 0.01 probability level, *NS* no significant difference *p* ≤ 0.05. *FI* full irrigation (DI_0%_) control received 100% of crop evapotranspiration (ETc), and DI_20%_ and DI_40%_, received 80% and 60% ETc, respectively; CB_0_, CB_10_ and CB_20_, CB mixture (1:1; w/w) at 0, 10, and 20 t ha^−1^, respectively

Saline calcareous soil amended with CB at the rate of 10 t ha^−1^ significantly (*P* ≤ 0.05) increased number of pods plant^−1^ (117.5%), SY plant^−1^ (47.0%), SI (19.5%), SHI (14.9%), BY (20.9%), and SY (40.0%), and WP (40.9%) that was further enhanced by 185.3% of number of pods plant^−1^, 126.5% of SY plant^−1^, 53.4% of SI, 29.2% of SHI, 56.4% of BY, 102.5% of SY, and 104.5% of WP in plants treated with 20 t CB ha^−1^, compared to unamended control planted in the same soil (Table 4).

There was a significant (*P* ≤ 0.05) effect of the DI × CB interaction on fenugreek yield and yield-related attributes and WP under saline calcareous soil. For example, the highest number of pods plant^−1^, SY plant^−1^, SI, SHI, BY and SY were obtained under FI × CB_20_ interaction with 465.3%, 532.1%, 133.0%, 53.4%, 95.2%, and 200.0%, respectively, higher than in plants of DI_40%_ × CB_0_ interaction (Table 4). Thus, this resulted in the lowest values for all these attributes across the two growing seasons when DI_40%_ × CB_0_ interaction was applied. The greatest WP values, representing 0.97 and 0.89 kg seed m^−3^, were obtained under DI_40%_ × CB_20_ and DI_20%_ × CB_20_ interactions, respectively, with 142.5% and 122.5% higher than FI × CB_0_ interaction, which recorded the lowest WP value (0.40 kg seed m^−3^) across the two growing seasons (Table 4).

### Oxidative stress indicators, osmoprotectants, and non-enzymatic antioxidants activity

**Fig. 1:**
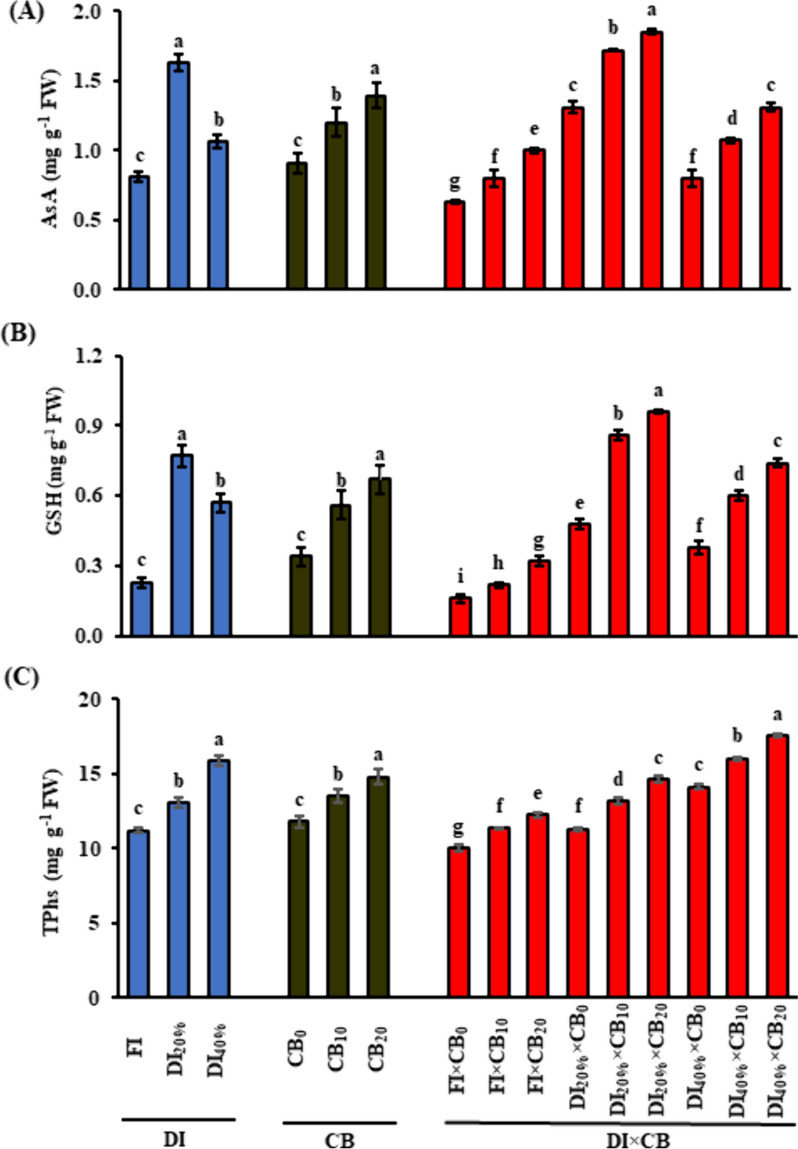
Effect of the application of CB mixture along with different DI levels on leaf (A) AsA; (B) GSH; and (C) TPhs of fenugreek (*Trigonella foenum-graecum* L.) plants grown under saline calcareous soil conditions. Vertical bar indicates mean ± standard error based on three replicates and different letters for each DI, CB, or DI × CB levels indicate significant differences according to the Duncan test (*P* ≤ 0.05). CB, compost and biochar; DI, deficit irrigation; AsA, ascorbic acid; glutathione; GSH; TPhs, total phenols. FI, full irrigation (DI_0%_) control received 100% of crop evapotranspiration (ETc), and DI_20%_ and DI_40%_, received 80% and 60% ETc, respectively; CB_0_, CB_10_ and CB_20_, CB mixture (1:1; *w/w*) at 0, 10 and 20 t ha^−1^, respectively

The current results elucidated that DI_20%_ or DI_40%_ treatments significantly (*P* ≤ 0.05) increased H_2_O_2_ by 5.1 or 9.9%, MDA by 19.1 or 46.8%, TSS by 43.8 or 71.9%, TPC by 17.2 or 36%, FProC by 15.8 or 24.2%, AsA by 101.2 or 30.9%, GSH by 234.8 or 147.8%, and TPhs by16.6 or 41.9%, respectively, compared to FI treatment (Table 5; Fig. 1). Compared to CB_0_ treatment, saline calcareous soil amended with 10 or 20 t CB ha^−1^ significantly (*P* ≤ 0.05) lowered H_2_O_2_ by 3.1% or 8.8% and MDA by 25.2 or 54.3%. However, it increased TSS by 25.8 or 103.2%, TPC by 10.6 or 22.6%, FProC by 7.2 or 15.3%, AsA by 31.9 or 52.7%, GSH by 64.7 or 97.1% and TPhs by 14.4 or 25.3%, respectively, compared to CB_0_ treatment (Table 5; Fig. 1).

**Table 5:** Effect of the application of CB mixture along with different DI levels on oxidative damage indices (H_2_O_2_ and MDA) and osmoprotectants (TSS, TPC, and FProC) of fenugreek (*Trigonella foenum-graecum* L.) plants grown under saline calcareous soil conditions in S_I_ and S_II_ growing seasons

Treatment		H_2_O_2_	MDA	TSS	TPC	FProC
		(µmol g^−1^FW)		(mg g^−1^ FW)		
Season		NS	NS	NS	NS	NS
S_I_		31.1 ± 0.12a	1.74 ± 0.14a	0.88 ± 0.07a	29.5 ± 0.9a	70.5 ± 1.5a
S_II_		30.4 ± 0.11a	1.74 ± 0.10a	0.90 ± 0.05a	29.3 ± 0.7a	70.1 ± 1.4a
DI		**	**	**	**	**
FI (DI_0%_)		29.3 ± 0.11c	1.41 ± 0.16c	0.64 ± 0.04c	25.0 ± 0.7c	62.0 ± 1.0c
DI_20%_		30.8 ± 0.12b	1.68 ± 0.11b	0.92 ± 0.06b	29.3 ± 0.7b	71.8 ± 1.1b
DI_40%_		32.2 ± 0.16a	2.07 ± 0.09a	1.10 ± 0.11a	34.0 ± 0.4a	77.0 ± 0.8a
CB (t ha^−1^)		**	**	**	**	**
0 (CB_0_)		32.0 ± 0.11a	2.34 ± 0.10a	0.62 ± 0.03c	26.5 ± 1.0c	65.5 ± 1.7c
10 (CB_10_)		31.0 ± 0.04b	1.75 ± 0.12b	0.78 ± 0.03b	29.3 ± 0.9b	70.2 ± 1.4b
20 (CB_20_)		29.2 ± 0.06c	1.07 ± 0.17c	1.26 ± 0.09a	32.5 ± 0.8a	75.1 ± 1.4a
DI × CB		**	**	**	**	**
FI (DI_0%_)	CB_0_	29.9 ± 0.02d	1.85 ± 0.06d	0.46 ± 0.02f	21.6 ± 0.2 g	56.6 ± 0.1 g
	CB_10_	29.4 ± 0.02d	1.57 ± 0.04f	0.62 ± 0.02e	24.6 ± 0.3f	62.5 ± 0.2f
	CB_20_	28.4 ± 0.02e	0.82 ± 0.08i	0.83 ± 0.01c	28.7 ± 0.3d	67.1 ± 0.1e
DI_20%_	CB_0_	31.8 ± 0.04bc	2.26 ± 0.04b	0.67 ± 0.02d	25.9 ± 0.2e	66.6 ± 0.3e
	CB_10_	31.2 ± 0.03c	1.72 ± 0.05e	0.84 ± 0.01c	29.3 ± 0.3d	71.7 ± 0.4d
	CB_20_	29.4 ± 0.03d	1.05 ± 0.10 h	1.23 ± 0.02b	32.5 ± 0.3c	77.3 ± 0.2b
DI_40%_	CB_0_	34.2 ± 0.04a	2.89 ± 0.03a	0.71 ± 0.01d	31.9 ± 0.1c	73.4 ± 0.4c
	CB_10_	32.4 ± 0.04b	1.96 ± 0.04c	0.88 ± 0.01c	33.9 ± 0.1b	76.6 ± 0.4b
	CB_20_	29.9 ± 0.07d	1.36 ± 0.06 g	1.71 ± 0.03a	36.3 ± 0.3a	80.9 ± 0.5a

Each value indicates mean ± standard error (*n* = 3). Means values in each column for DI, CB, or DI × CB levels followed by the same lower-case letter in each column are not significantly different according to the Duncan test (*P ≤* 0.05). *CB* compost and biochar, *DI* deficit irrigation, *H*_*2*_*O*_*2*_ hydrogen peroxide, *MDA* malondialdehyde, *TSS* total soluble sugars, *TPC* total protein content, *FProC* free proline content; S_I_ and S_II_, 2021–2022 and 2022–2023 growing seasons, respectively. * and **, differences at *p* ≤ 0.05 and 0.01 probability level; *NS* no significant difference *P* ≤ 0.05. *FI* full irrigation (DI_0%_) control received 100% of crop evapotranspiration (ETc), and DI_20%_ and DI_40%_, received 80% and 60% ETc, respectively; CB_0_, CB_10_ and CB_20_, CB mixture (1:1; w/w) at 0, 10, and 20 t ha^−1^, respectively

The DI × CB interaction also impacted oxidative stress indicators, osmoprotectants, and antioxidant activity of fenugreek plants raised in calcareous, saline soil over the two seasons. On average of the two seasons, the highest H_2_O_2_ (34.2 nmol g^−1^ FW) and MDA (2.89 µmol g^−1^ FW) levels in fenugreek leaves were recorded in the DI_40%_ × CB_0_ treatment, where the lowest H_2_O_2_ (28.4 nmol g^−1^ FW) and MDA (0.82 µmol g^−1^ FW) levels were observed in FI × CB_20_ treatment (Table 5). Likewise, the highest levels of TSS, TPC, FProC, and TPhs were also noticeable when DI_40%_ × CB_20_ treatment was applied (Table 5). However, leaves of fenugreek plants treated with DI_20%_ × CB_20_ accumulated higher AsA and GSH levels than DI_20%_ × CB_0_ or DI_20%_ × CB_10_ treatment (Fig. 1).

### Enzymatic antioxidants and DPPH RSA

**Fig. 2:**
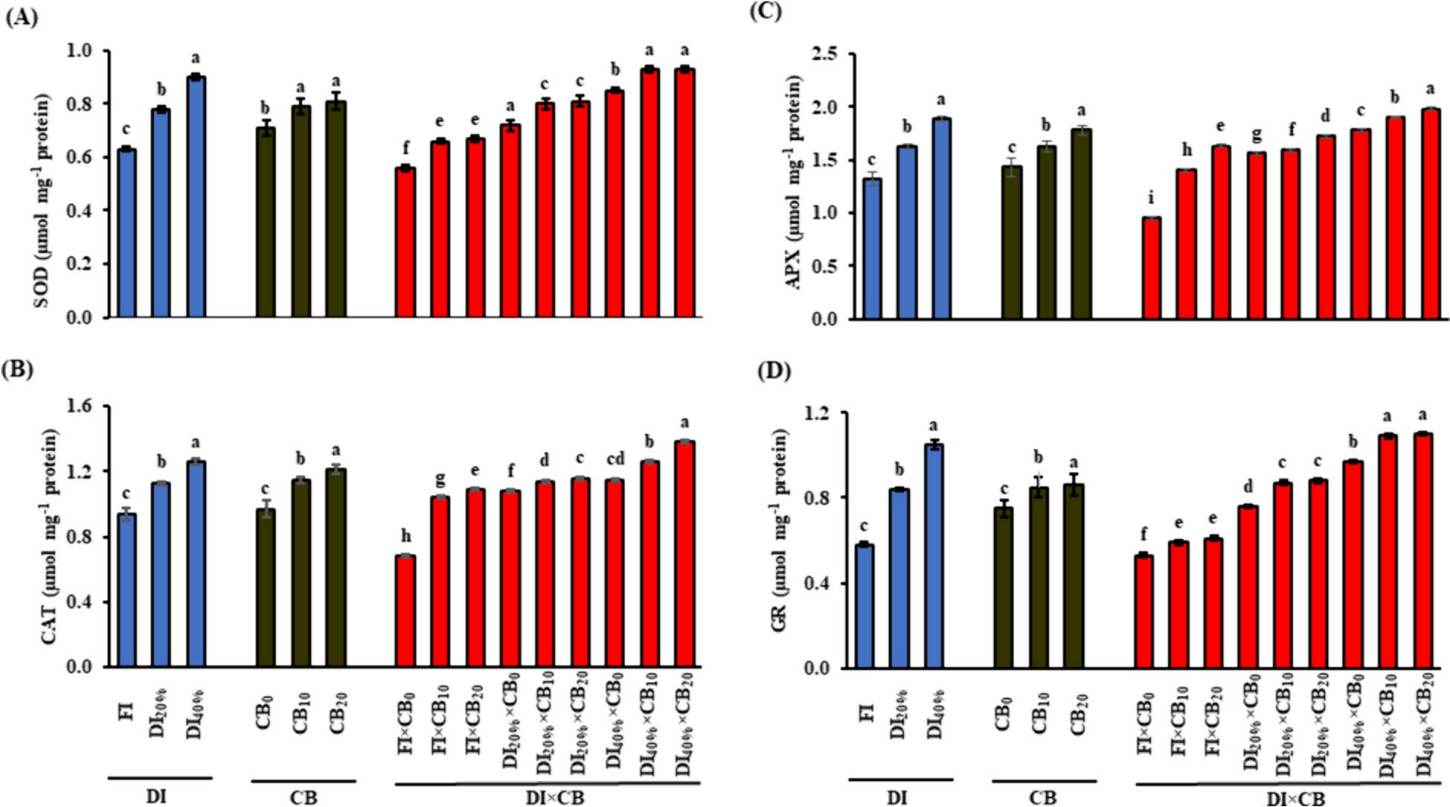
Effect of the application of CB mixture along with different DI levels on enzymatic activity of (A) SOD; (B) CAT; (C) APX; and (D) GR of fenugreek (*Trigonella foenum-graecum* L.) plants grown under saline calcareous soil conditions. Vertical bar indicates mean ± standard error based on three replicates and different letters for each DI, CB, or DI × CB levels indicate significant differences according to the Duncan test (*P ≤* 0.05). CB, compost and biochar; DI, deficit irrigation; SOD, superoxide dismutase; CAT, catalase; APX, ascorbate peroxidase; GR, glutathione reductase. FI, full irrigation (DI_0%_) control received 100% of crop evapotranspiration (ETc), and DI_20%_ and DI_40%_, received 80% and 60% ETc, respectively; CB_0_, CB_10_ and CB_20_, CB mixture (1:1; w/w) at 0, 10 and 20 t ha^−1^, respectively

**Fig. 3:**
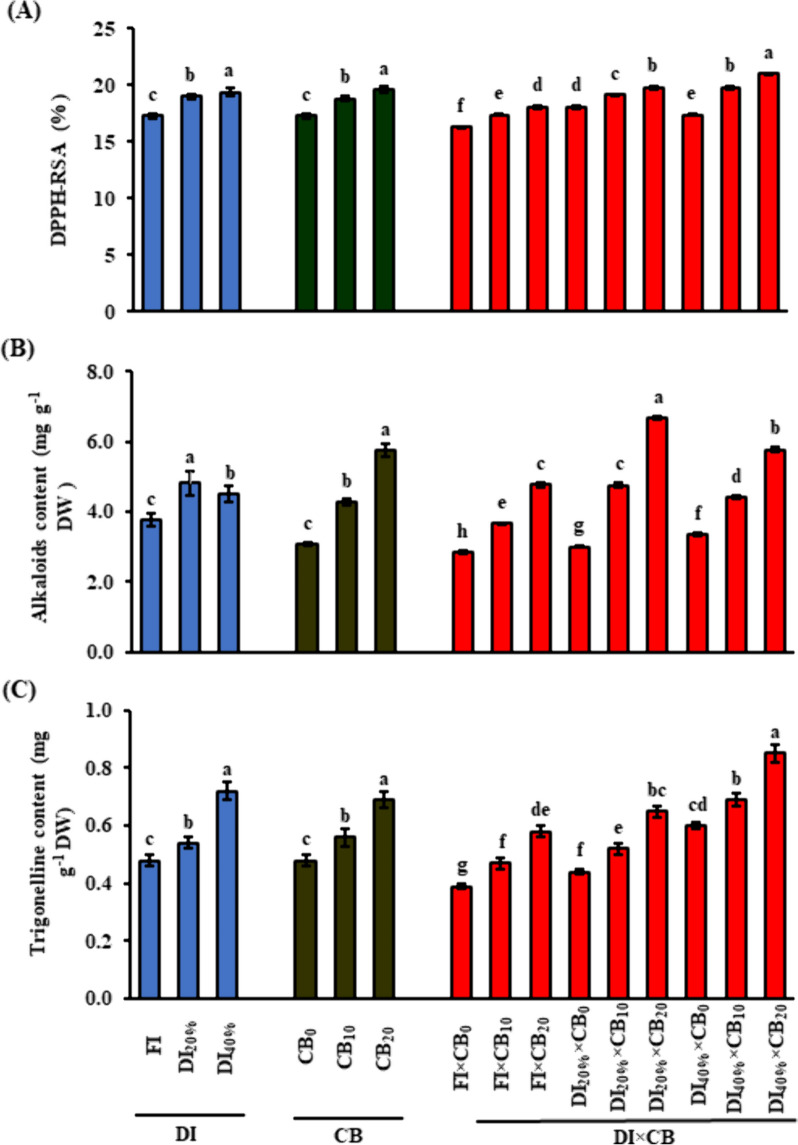
Effect of the application of CB mixture along with different DI levels on leaf (A) DPPH RSA; and seed (B) alkaloids and (C) trigonelline contents of fenugreek (*Trigonella foenum-graecum* L.) plants grown in saline calcareous soil. Vertical bar indicates mean ± standard error based on three replicates and different letters for each DI, CB, or DI × CB levels indicate significant differences according to the Duncan test (*P ≤* 0.05). CB, compost and biochar; DPPH, 2,2-diphenyl-1- picrylhydrazyl; DPPH RSA, DPPH radical-scavenging activity; DI, deficit irrigation; DW, dry weight; FI, full irrigation (DI_0%_) control received 100% of crop evapotranspiration (ETc), and DI_20%_ and DI_40%_, received 80% and 60% ETc, respectively; CB_0_, CB_10_ and CB_20_, CB mixture (1:1; w/w) at 0, 10 and 20 t ha^−1^, respectively

In response to DI_20%_ and DI_40%_, plants significantly (*P* ≤ 0.05) increased the activity of SOD by 23.8 and 42.9% (Fig. 2A), CAT by 20.2 and 34.0% (Fig. 2B), APX by 23.5 and 43.2% (Fig. 2C), GR by 44.8 and 81.0% (Fig. 2D), respectively, compared to FI treatment. Similarly, DPPH RSA was significantly (*P* ≤ 0.05) increased by 9.8% in DI_20%_ and 12.1% in DI_40%_ when compared to FI (Fig. 3A). Moreover, an increment was noticed in the activation of the aforementioned antioxidative enzymes and DPPH RSA when the saline calcareous soil was amended with 10 or 20 t CB ha^−1^ (Figs. 2 and 3A). In fenugreek plants treated with CB_10_, the activities of SOD, CAT, APX and GR were 11.3, 18.6, 14.0, and GR 13.3% greater than in CB_0_, respectively. The same enzymes were also higher by 14.1, 24.7, 24.5, and 14.7%, respectively, than plants treated with CB_20_. The antioxidant activity of DPPH RSA was increased by 8.7 and 13.3% in fenugreek plants treated with CB_10_ or CB_20_, respectively, compared to that in CB_0_.

The DI × CB interaction significantly (*P* ≤ 0.05) increased the activity of antioxidant indicators of the fenugreek plant under saline calcareous soil conditions in both seasons. This was evidenced by the higher activities of SOD (66.1%; Fig. 2A), CAT (102.9%; Fig. 2B), APX (108.4%; Fig. 2C), GR (107.5%; Fig. 2D), and DPPH RSA (28.8%; Fig. 3A) in plants under DI_40%_ × CB_20_ interaction than those obtained under FI × CB_0_ interaction.

### Seed alkaloids and trigonelline content

Pooled data from the two years showed that the effects of DI strategy, CB rate, and their interaction on the total seed alkaloids and trigonelline contents were significant (*P* ≤ 0.05; Fig. 3B & C). Once increasing drought stress severity to DI_20%_ and DI_40%_ levels, the total seed alkaloids, and trigonelline content significantly (*P* ≤ 0.05) increased by 27.9 and 19.9%, and 12.5 and 50.0%, respectively, compared to those that do not suffer from drought stress. The total seed alkaloids and trigonelline contents in dry fenugreek seeds under CB_20_ treatment were 5.74 and 0.69 mg g DW^−1^, (approximately 87.0 and 43.8%, respectively) greater than that under unamended (CB_0_) treatment.

The DI × CB interaction significantly (*P* ≤ 0.05) affected the total seed alkaloids and trigonelline contents (Fig. 3B & C). Compared to FI (DI_0%_)-treated plants, stressed plants cultivated in saline calcareous soil at the rate of 20 t ha^−1^ CB elevated the total seed alkaloids content by 134.9% and 103.2% and trigonelline contents by 66.7% and 117.9% in response to moderate to severe drought stress conditions, respectively (Fig. 3B & C).

### Leaf mineral content

Throughout the two growing seasons of fenugreek, the DI level, CB rate, and their interaction had substantial (*P* ≤ 0.05) impacts on the leaf mineral contents of fenugreek grown in the tested saline calcareous soil (Table 6). In general, plants subjected to DI_20%_ or DI_40%_ levels showed reduced nutrient uptake of N, P, K^+^ and Ca^2+^ as well as K^+^/Na^+^ ratio by 8.6 or 18.5%, 17.6 or 39.5%, 2.9 or 5.0%, 23.5 or 42.6% and 1.7 or 6.6%, respectively, compared to fully irrigated plants (Table 6). In contrast, more increment was noted by 2.2 to 5.9% in Na^+^ uptake in response to drought stress and the intensity increased from DI_20%_ to DI_40%_ compared to the non-stressed treatment (Table 6).

**Table 6:** Effect of the application of CB mixture along with deficit irrigation (DI) levels on leaf elemental (nitrogen; N, phosphorus; P, potassium; K^+^, sodium; Na^+^, and calcium; Ca^2+^) status of fenugreek (*Trigonella foenum-graecum* L.) plants grown under saline calcareous soil conditions in S_I_ and S_II_ growing seasons

Treatment		N	P	K^+^	Na^+^	Ca^2+^	K^+^/Na^+^ ratio
		(%)	(mg g^−1^ DW)				
Season		*	*	**	**	*	NS
S_I_		3.93 ± 0.05a	5.21 ± 0.28b	22.7 ± 0.21b	13.5 ± 0.56b	10.0 ± 0.64b	1.77 ± 0.08a
S_II_		3.66 ± 0.12b	5.23 ± 0.30a	24.2 ± 0.23a	14.4 ± 0.49a	11.2 ± 0.58a	1.75 ± 0.07a
DI		**	**	**	**	**	**
FI (DI_0%_)		4.17 ± 0.06a	6.69 ± 0.19a	24.1 ± 0.31a	13.6 ± 0.73b	13.6 ± 0.57a	1.81 ± 0.08a
DI_20%_		3.81 ± 0.08b	5.51 ± 0.24b	23.4 ± 0.29b	13.9 ± 0.44b	10.4 ± 0.63b	1.78 ± 0.10a
DI_40%_		3.40 ± 0.12c	4.05 ± 0.27c	22.9 ± 0.30c	14.4 ± 0.85a	7.8 ± 0.56c	1.69 ± 0.10b
CB (t ha^−1^)		**	**	**	**	**	**
0 (CB_0_)		3.48 ± 0.13c	4.40 ± 0.32c	22.4 ± 0.25c	17.4 ± 0.44a	8.2 ± 0.61c	1.31 ± 0.04c
10 (CB_10_)		3.82 ± 0.09b	5.41 ± 0.28b	23.4 ± 0.23b	13.4 ± 0.25b	10.2 ± 0.64b	1.80 ± 0.03b
20 (CB_20_)		4.07 ± 0.07a	6.45 ± 0.25a	24.5 ± 0.27a	11.1 ± 0.17c	13.4 ± 0.63a	2.22 ± 0.02a
DI × CB		NS	NS	NS	**	NS	**
FI (DI_0%_)	CB_0_	3.93 ± 0.07a	5.92 ± 0.21a	23.0 ± 0.28a	15.0 ± 0.08c	11.2 ± 0.47a	1.54 ± 0.01e
	CB_10_	4.18 ± 0.05a	6.64 ± 0.17a	24.1 ± 0.45a	14.7 ± 0.24c	13.2 ± 0.52a	1.65 ± 0.01d
	CB_20_	4.42 ± 0.07a	7.52 ± 0.21a	25.1 ± 0.53a	11.0 ± 0.28f	16.2 ± 0.58a	2.25 ± 0.02a
DI_20%_	CB_0_	3.52 ± 0.16a	4.41 ± 0.18a	22.4 ± 0.42a	18.0 ± 0.23b	7.8 ± 0.38a	1.24 ± 0.02f
	CB_10_	3.87 ± 0.06a	5.61 ± 0.14a	23.4 ± 0.34a	12.4 ± 0.26e	9.9 ± 0.50a	1.88 ± 0.01b
	CB_20_	4.02 ± 0.08a	6.52 ± 0.25a	24.6 ± 0.33a	11.2 ± 0.23f	13.4 ± 0.55a	2.21 ± 0.05a
DI_40%_	CB_0_	2.99 ± 0.24a	2.87 ± 0.19a	21.9 ± 0.48a	19.1 ± 0.36a	5.5 ± 0.34a	1.15 ± 0.02 g
	CB_10_	3.41 ± 0.16a	3.98 ± 0.18a	22.8 ± 0.27a	13.2 ± 0.24d	7.4 ± 0.51a	1.73 ± 0.02c
	CB_20_	3.79 ± 0.07a	5.31 ± 0.25a	23.9 ± 0.44a	11.2 ± 0.39f	10.6 ± 0.44a	2.19 ± 0.04a

Each value indicates mean ± standard error (*n* = 3). Means values in each column for DI, CB, or DI × CB levels followed by the same lower-case letter in each column are not significantly different according to the Duncan test (*P ≤* 0.05). *CB* compost and biochar, *DI* deficit irrigation, *N* nitrogen, *P *phosphorus, *K*^+^ potassium, *Na*^+^ sodium, *Ca*^2+^ calcium; S_I_ and S_II_, 2021–2022 and 2022–2023 growing seasons, respectively. * and **, differences at *p* ≤ 0.05 and 0.01 probability level; *NS* no significant difference *P* ≤ 0.05. *FI* full irrigation (DI_0%_) control received 100% of crop evapotranspiration (ETc), and DI_20%_ and DI_40%_, received 80% and 60% ETc, respectively; CB_0_, CB_10_ and CB_20_, CB mixture (1:1; w/w) at 0, 10, and 20 t ha^−1^, respectively

Compared to the unamended treatment, nutrients uptake of fenugreek plants grown in saline calcareous soil amended with CB_10_ or CB_20_ treatments were significantly (*P* ≤ 0.05) increased by 9.8 or 17.0% for N, 17.6 or 46.6% for P, 4.5 or 9.4% for K^+^, 24.4 or 63.4% for Ca^2+^, and 37.4 or 69.5% for K^+^/Na^+^ ratio; however, leaf Na^+^ content was reduced by 23.0 or 36.2% respectively (Table 6). Compared with DI_40%_ × CB_0_ interaction, Na^+^ content was significantly (*P* ≤ 0.05) lower by 42.4%, but 95.7% higher in the K^+^/Na^+^ ratio in leaf tissues of fenugreek plants under FI × CB_20_ interaction, which was similar to that in plants treated with the combinations of DI_20%_ × CB_20_ and DI_40%_ × CB_20_ (Table 6).

## Discussion

This study illustrated the positive impact of CB mixture as a soil amendment to improve soil physiochemical characteristics, soil-water interactions, and nutrient retention. This aligns with previous research indicating enhanced physical, chemical, and biological functions in soils following CB incorporation [35, 37, 78]. Consequently, properties, such as TP, WHC and BD, OM, and NPK content show improvement across various soil types with CB application. Our results underscore the potential of CB as a crucial component in water management aimed at boosting the growth, development, and productivity of fenugreek plants cultivated in saline calcareous soil under conditions of water scarcity.

The diminished productivity observed in non-CB-amended soil in this study could be due to the adverse effects induced by DI on various aspects of fenugreek growth and physiology. These effects include compromised root-shoot growth parameters such as PH, leaf number plant^−1^, branch number plant^−1^, root length, and plant dry matter, as well as reductions in leaf tissue water content (measured by MSI and RWC) and leaf photosynthetic efficiency (assessed by *F*_*v*_*/F*_*m*_, *F*_*v*_*/F*_*0*_ and PI) under conditions of saline calcareous soil (Tables 2 and 3). Due to DI stress in saline calcareous soil, similar reductions in growth, yield, and related components of fenugreek (e.g., increased flower or pod abortion, reduced seed sets, fewer seeds pod^−1^, and diminished seed size) have been previously reported [8, 79–82]. These detrimental effects are likely attributable to the elevated soil ECe and pH levels, coupled low WHP, TP, and water retention capacity (Table 1). These soil conditions impede root proliferation, limit aeration, and hinder water and nutrient uptake by plant roots, ultimately leading to reduced productivity.

In addition, the decreased SHI of fenugreek under DI stress, indicating limited allocation of photo-assimilates to seeds, likely contributes to the reduction in SY [83]. However, the application of CB_10_ and CB_20_ to saline calcareous soil resulted in substantial increases in yield and its associated attributes of fenugreek plants compared to those in CB_0_-amended soil under full- or DI regimes.

The combined application of CB_20_ and DI_20%_ effectively mitigated the adverse effects of water deficit on fenugreek growth, resulting in notable enhancement in PH, leaf count plant^−1^, branch count plant^−1^, pod count plant^−1^, SY plant^−1^, and BY with values closely resembling those observed in plants treated with FI × CB_20_. Compared to untreated soils (CB_0_), the incorporation of 10 or 20 t CB ha^−1^ improved fenugreek growth, SY, and related components. The observed growth enhancement may be linked to increased CB decomposition and soil nutrient mineralization [84]. In addition, CB application could enhance soil structure, nutrients supply, and humic acid provision, thereby enhancing soil’s capacity to retain both nutrients and water [12, 85], a phenomenon supported by our findings.

The enhanced SY observed following the application of CB_10_ and CB_20_ can be attributed to several factors. Firstly, the increase in soil OM and fertility facilitates greater availability of water and nutrients for plant uptake [12, 85]. Secondly, the application of CB induces significant modifications in soil physico-chemical properties, including enhanced soil structure, reduced soil Na^+^ content, increased root proliferation [10], and improved water and nutrient-uptake efficiency (Table 1), all contributing to enhanced seed production. Lastly, the decrease in soil ECe and pH resulting from CB application promotes the uptake of certain micronutrients and aids in the regulation of the soil solution ionic balance, further supporting SY enhancement [26, 86].

RWC and MSI have emerged as significant indicators of drought tolerance and cellular membrane integrity, reflecting the extent of oxidative stress. Similar to other reports [10, 87], MSI and RWC exhibited a decline in fenugreek plants subjected to DI stress (Table 3), a change likely stemming from reduced levels of endogenous abscisic acid, a key regulator of stomatal closure [88]. The preservation of water transport due to the turgidity of mesophyll cells and leaf tissue thickness might be another reason of decline [81]. However, CB application can ameliorate plant water status, including RWC and MSI, even in the presence of limited soil water moisture. This beneficial effect can be attributed to the capacity of compost and/or biochar to enhance soil water retention, thereby increasing AW content in plants [89, 90]. This observation aligns with findings by Abd El-Mageed et al. [12], who noted that the combined CB application augmented water content in plant tissues grown in salt-affected soil under soil water deficit.

CB serves as an effective carrier and source of essential nutrients, including N, P, K^+^ and Ca^2+^, enriching the soil solution and reducing rhizospheric leaching [91]. Its porous organic nature enables CB to enhance the RWC and MSI of fenugreek [92], thereby enhancing water retention capacity, overall aeration porosity, and nutrient bioavailability within the soil. Consequently, the incorporation of CB can lead to reduced irrigation water demands while simultaneously improving soil conditions for plant growth [10].

Plants employ different adaptations to cope with decreased photosynthetic activity. One strategy involves adjusting pigment composition, wherein plants may alter the ratio of chlorophyll *a* and *b* to optimize light absorption [93]. In response to environmental stress, plants often close stomata to minimize water loss, thereby limiting the availability of CO_2_ available for photosynthesis and subsequent dry matter accumulation [79]. Furthermore, plants activate their antioxidant defense system, producing antioxidants such as AsA and GSH, to alleviate oxidative damage induced by abiotic stress [94, 95].

Similar to lupine plants thriving in saline calcareous soil [10], drought imposition resulted in a significant decrease in the relative chlorophyll content (SPAD_chlorophyll_) and photosynthetic efficiency (*F*_*v*_*/F*_*m*_, *F*_*v*_*/F*_*0*_ and PI) in stressed fenugreek plants compared to their non-stressed counterparts under well-watered conditions. The diminished enzyme activity under drought stress leads to a reduction in chlorophyll production [79]. In addition, drought stress can instigate the disruption of chloroplast membrane integrity, consequently promoting the degradation or breakdown of chlorophyll molecules. This degradation contributes to the overall chlorophyll content in plant cells [27]. Therefore, the decline in chlorophyll content may be attributed to the generation of reactive oxygen species (ROS) and the increased activities of the chlorophyll-degrading enzymes [25, 29].

The decrease in the number of leaves plant^−1^ induced by drought stress significantly contributes to the reduction in crop yield by impeding the process of photosynthesis [20]. Drought-induced reduction in leaf area commonly occurs as a mechanism to mitigate water loss through canopy transpiration [96]. Our observations align with previous findings [10, 83], indicating lower plant water status, decreased photosynthetic pigments, reduced performance in photosynthetic parameters such as PI and *F*_*v*_*/F*_*m*_, and diminished leaf area under drought stress conditions. These factors collectively contribute to reductions in RL, shoot biomass (e.g., OM) and overall SY and its associated attributes.

Enhancing WP stands as a critical important strategy in addressing the global water scarcity challenge, focusing on maximizing crop yields per unit of water consumed. Particularly in irrigated agricultural settings, the emphasis on improving WP outweighs the priority of increasing yield potential per unit area for growers [97]. Our field experiments revealed that fenugreek plants subjected to severe drought stress (DI_40%_) increased WP (Table 3). These plants demonstrated resilience to water deficits by achieving significantly higher yields while utilizing less irrigation water, highlighting their potential in water-saving cultivation practices [79].

Fenugreek plants cultivated in saline calcareous soil, amended with either 10 or 20 t CB ha^−1^, exhibited significant increases in WP by 40.9%, and 104.5%, respectively, compared to non-CB-amended plants. Notably, the application of DI_40%_ × CB_20_ demonstrated the highest WP, reaching 142.5%. Furthermore, DI_20%_ × CB_20_ treatment conserved an additional 20% of water while enhancing WP to 122.5% compared to FI without CB, in agreement with findings from previous studies [89]. Obadi et al. [98] also observed enhanced WP in drought-stressed pepper plants supplemented with a CB mixture (2:2).

Drought stress typically leads to oxidative damage, evidenced by increased levels of MDA and accumulation of ROS, such as H_2_O_2_, in fenugreek leaves. These phenomena potentially contribute to membrane damage and lipid peroxidation in plant cells [80, 99], ultimately affecting fenugreek leaf water relations.

The application of CB resulted in an increase in the accumulation of osmolytes, such as TSS, TPC and FProC (Table 5), along with non-enzymatic antioxidants including AsA, GSH and TPhs (Fig. 1). This suggests that these physio-metabolic adaptive mechanisms could enhance tolerance to salinity and drought stresses [100, 101]. Previous studies have indicated that organic osmoprotectants present in CB-treated fenugreek plants are associated with the osmotic regulation, safeguarding cellular membrane integrity under severe DI conditions [12]. Essential ROS-scavenging mechanisms in plants involve enzymatic activities of SOD, CAT, APX and GR, and DPPH radical-scavenging activity (DPPH RSA). Hence, the regulation of enzymatic components within the antioxidant machinery is crucial for maintaining the “delicate” balance between the production and elimination of ROS and MDA levels in stressed fenugreek plants [102]. The observed enhanced growth and yield, associated with elevated GSH levels, could be attributed to the critical role of GSH in mitigating ROS-induced damage and enhancing tolerance in fenugreek and other plant species [79, 100, 103].

Trigonelline, a pyridine alkaloid compound present in fenugreek and other plant species [104], serves as an important osmoregulatory metabolite, playing a key role in regulating osmotic pressure induced by drought [96]. Studies have shown an elevation in trigonelline concentration in fenugreek and lupin seeds under salinity and drought conditions [10, 83, 105]. In our investigation, DI increased trigonelline levels, yet the application of CB not only reduced trigonelline and total alkaloid contents but also alleviated the adverse effects of DI stress on fenugreek plants. The high accumulation of secondary metabolites, such as trigonelline, in environmentally stressed fenugreek seeds likely serves to counteract excessive production of ROS and the resulting photoinhibition damage [106, 107]. Moreover, plants exposed to abiotic stresses accelerate nitrate accumulation and hinder protein biosynthesis in plant tissues [108], facilitating their incorporation into secondary metabolites, such as alkaloids [108, 109].

The reduction in nutritional status of N, P, K^+^, and Ca^2+^ in fenugreek plants exposed to DI in saline calcareous soil may be attributed to the constrained kinetics of nutrient uptake, closely linked to diminished soil moisture levels [110]. As documented previously [111], the accumulation of excessive Na^+^ ions in the cells of fenugreek plants grown in saline calcareous soil disrupts ionic balance and restricts the uptake of other essential nutrients such as N, P, K^+^, and Ca^2+^. However, the application of CB positively influences ionic equilibrium and enhances nutrient uptake under saline calcareous soil conditions. CB serves as an additional element source of OM, N, P, and K^+^, directly augmenting nutrient levels in the soil. In leaf tissues of DI-stressed fenugreek, the incorporation of 10 or 20 t, CB ha^−1^ increased the concentrations of N, P, K^+^, and Ca^2+^ as well as the K^+^/Na^+^ ratio while decreasing Na^+^ ions concentration (Table 6). Notably, N availability was significantly improved with the provision of irrigation and CB [112]. The decrease in Na^+^ ion concentration in fenugreek leaves can be attributed to the application of CB, acting as a biochar-containing soil amendment with a high affinity for adsorbing Na^+^ ions on its surface, thereby facilitating Na^+^ leaching from the plant rhizosphere and promoting the restoration of saline soil conditions [113].

Singh et al. [114] suggest that maintaining a high K^+^/Na^+^ ratio during drought and/or salinity stress may represent a plant’s adaptive response to uphold cytosolic cation balance, thereby preserving cellular osmotic pressure and turgor. In our investigation, the application of CB resulted in elevated levels of Ca^2+^ and K^+^ compared to Na^+^. Consequently, the K^+^/Na^+^ ratio substantially increased in fenugreek plant tissues under DI stress conditions. During environmental stresses, it is imperative to sustain the structural and functional integrity of plant membranes with adequate levels of K^+^ and Ca^2+^ [100, 115]. Similar observations regarding the restoration of ionic homeostasis and enhancement of nutrient profiles under drought stress through CB application have been reported in eggplant [12], fenugreek [34], and sainfoin [99].

It is worth mentioning that microbial processes play a crucial role in enhancing the availability and accessibility of essential nutrients crucial for sustaining plant health [116]. CB, as a C-rich amendment, has the potential to serve as a source of nutrients and habitat for soil microorganisms. This, in turn, can contribute to the stabilization of soil structure and the promotion of beneficial rhizospheric microorganisms, including N-fixing bacteria, thereby bolstering plant resilience to environmental stress [117, 118]. Future research avenues could explore the impact of CB application on the diversity of soil microbial communities, their ecological functions, soil enzyme activities, and the functional genes associated with improved crop yield and quality, all through the lens of soil microbial dynamics.

## Conclusions

The present study indicated that the application of CB mixture to saline calcareous soils could have many benefits as a soil ameliorant, even under DI stress. Soil application of 10 or 20 t h^−1^ of CB led to improvements in soil physical (BD, WHP, UP, AW content and θ_Fc_), chemical (acidity, ECe, OM, N, and P contents) properties, and potentially beneficial to the rhizosphere microorganisms. These contributed to improvements in succulence (RWC and MSI), quantum efficiency of PSII (leaf greenness, chlorophyll *a* and PI) and nutritional homeostasis (high N, P, K^+^, Ca^+2^, and K^+^/Na^+^ ratio and lower Na^+^) in fenugreek leaves. This was also supported by the increase of osmolytes (TSS, TPC, and FProC), non-enzymatic (AsA, GSH, and TPhs), and enzymatic (SOD, CAT, APX, and GR) antioxidant activities, and DPPH RSA to scavenge ROS (H_2_O_2_ and MDA) under drought stress conditions. This suggests that these physio-metabolic adaptative mechanisms can improve stress tolerance in fenugreek. The addition of 20 t ha^−1^ CB mixture to saline calcareous soil under moderate water deficit (DI_20%_) could save up to 20% of the water applied yielding higher quality (trigonelline and total alkaloid contents) and WP. Thus, this could be commercially marketed for producing fenugreek crops in saline calcareous soil when irrigation water is limited. It is recommended to water at 80% ETc, combined with 20 t ha^‒1^ CB, in arid agricultural areas to optimize water use and maintain crop health.

**Tables**.

### Supplementary Information

Supplementary Material 1.

## Data Availability

All datasets generated for this study are included in the article/Supplementary Materials.
