# Supplementary material for: Incorporation of compost and biochar enhances yield and medicinal compounds in seeds of water-stressed Trigonella foenum-graecum L. plants cultivated in saline calcareous soils
Source: BMC Plant Biol. 2024 Jun 12;24:538. doi: 10.1186/s12870-024-05182-6 (PMC11167906; doi:10.1186/s12870-024-05182-6)
Supplement: Supplementary file 1 — Supplementary Material 1. [file 12870_2024_5182_MOESM1_ESM.pdf]

Supplementary Materials

Incorporation of Compost and Biochar Enhances Yield and Medicinal Compounds in Seeds of Water-stressed *Trigonellia foenum-graecum* L. Plants Cultivated in Saline Calcareous Soils

Ahmed Shaaban[1], Khaulood A. Hemida[2], Taia A. Abd El-Mageed[3], Wael M. Semida[4], Synan F. AbuQamar[5,*], Mohamed T. El-Saadony[6], Omar A.A.I. Al-Elwany[4] and Khaled A. El-Tarabily[5]

[1]Agronomy Department, Faculty of Agriculture, Fayoum University, Fayoum, 63514, Egypt

[2]Botany Department, Faculty of Science, Fayoum University, Fayoum, 63514, Egypt

[3]Soil and Water Department, Faculty of Agriculture, Fayoum University, Fayoum, 63514, Egypt

[4]Horticulture Department, Faculty of Agriculture, Fayoum University, Fayoum, 63514, Egypt

[5]Department of Biology, College of Science, United Arab Emirates University, Al Ain, 15551, United Arab Emirates

[6]Department of Agricultural Microbiology, Faculty of Agriculture, Zagazig University, Zagazig, 44519, Egypt

*** Correspondence**:

Synan F. AbuQamar (sabuqamar@uaeu.ac.ae)

Table S1. Physicochemical properties of CB mixture.

**Property**	**Unit**	**Value**
Organic C	g kg^{-1}	396
ECe	dS m^{-1}	4.62
BD	g cm^{-3}	0.86
WHC	%	26.23
Water content	v/v	25.34
pH		7.42
Cation exchange capacity	$cmol^{+}$ kg^{-1}	46.50
N	%	1.83
P	g kg^{-1}	2.89
K^{+}		2.54
Ca^{2+}		2.51
Zn	mg kg^{-1}	66.72
Fe		76.32
Mn		165.6
Cu		20.21

CB, compost and biochar; C, carbon; ECe, electrical conductivity of soil extract; BD, bulk density; WHC, water holding capacity; N, nitrogen; P, phosphorus; K^{+}, potassium; Ca^{2+}, calcium; Zn, zinc; Fe, iron; Mn, Manganese; Cu, copper.

Table S2. Mean monthly air temperature, average relative humidity (RH_{avg}), and wind speed (WS) at Fayoum, Egypt, as the average for 2021-2022 (SI) and 2022-2023 (SII) seasons.

Month	Main air temperature (°C)						RH_{avg} (%)		WS (m s^{-1})	
	T_{max}		T_{min}		T_{avg}					
	SI	SII	SI	SII	SI	SII	SI	SII	SI	SII
October	28.5	29.3	16.9	17.5	22.7	23.4	42.9	43.5	2.13	2.09
November	27.8	28.4	16	15.2	21.9	21.8	44.7	43.7	1.96	2.00
December	20.8	21.2	12.4	12.6	16.6	16.9	43.2	44	2.02	1.98
January	20.2	20.8	9.4	9.6	14.8	15.2	42.3	43.7	3.00	2.90
February	22.5	21.9	8.7	9.1	15.6	15.5	41.2	43	3.23	3.19
March	26.8	27.8	13.1	12.7	20.0	20.3	38.8	37.8	2.16	2.28
April	29.4	31	17	16.2	23.2	23.6	38	37.2	2.00	1.96

$T_{max/min/avg}$ = maximum, minimum, and average temperatures; RH_{avg}, average relative humidity; WS, wind speed
